# Clinical Usefulness of a Modified Mohs' Technique and Topical Application of Zinc Oxide Powder for Treating Skin Infiltration Caused by Unresectable Malignant Tumors

**DOI:** 10.1089/pmr.2020.0107

**Published:** 2021-06-14

**Authors:** Masaru Arima, Kenta Saito, Tamaki Maeda, Hidehiko Fukushima, Yohei Iwata, Kazumitsu Sugiura

**Affiliations:** ^1^ Department of Dermatology, Fujita Health University School of Medicine, Aichi, Japan.; ^2^ Department of Dermatology, Fujita Health University BANTANE Hospital, Aichi, Japan.

**Keywords:** arterial embolization, modified Mohs', technique, palliative care, zinc oxide starch powder

## Abstract

***Background:*** Infiltrative lesions of the skin caused by unresectable malignant tumors reduce the quality of life of patients significantly due to the presence of bleeding, exudate, pain, and/or malodor.

***Objective:*** We compared the efficacy of a modified Mohs' technique and topical application of a starch powder containing zinc oxide as palliative treatments for skin lesions caused by unresectable tumors in our hospital.

***Design:*** This is a retrospective study.

***Settings/Subjects:*** This study included nine patients who were treated for skin-infiltrating lesions caused by unresectable malignant tumors at our hospital in Japan from April 2008 to December 2019.

***Measurements:*** Mohs' paste or zinc oxide powder (50%) was applied to the infiltrative skin lesions. Arterial embolization was performed before the application of the Mohs' paste for patients at risk for arterial hemorrhage. Patients were evaluated for pain, tumor size, bleeding, wound exudate, and malodor.

***Results:*** Both treatments were useful for alleviating symptoms, such as tumor size, local bleeding, malodor, and exudate in patients with unresectable malignant tumors. Pain was reduced in patients treated with Mohs' paste for 1 hour as compared with those treated for 24 hours.

***Conclusions:*** Effective management of skin infiltrative lesions can be achieved by using a modified Mohs' technique, topical application of starch powder containing zinc oxide, and arterial embolization to reduce the vascularization of the tumors.

## Introduction

Skin-infiltrating lesions caused by unresectable malignant tumors may grow and protrude superficially or create an ulcer. The patient's quality of life (QOL) is severely impaired by the presence of bleeding, exudate, and malodor from these lesions. Mohs' chemosurgery is a method that was developed by F.E. Mohs, a surgeon in the United States.^1^ This method utilizes the corrosive properties of zinc chloride, which is the main component, and involves repeated fixation and excision until the tumor is completely removed (based on the pathological findings of the excised tissue).^1,2^ Originally intended to be a curative treatment, it has recently been used for the purpose of improving the QOL for patients with unresectable malignant tumors.^3–5^

A main disadvantage of the Mohs' technique is the severe pain and ulceration caused by the adherence of the paste to the normal skin surrounding the tumor. Therefore, with an emphasis on pain relief, we reduced the application time of the paste from 24 to <1 hour. Furthermore, we compared the abilities of zinc oxide powder and Mohs' paste in reducing skin tumor size, local bleeding, malodor, and exudates. To our knowledge, this is the first report that utilized a modified Mohs' technique with the topical application of zinc oxide powder as part of palliative treatment for skin lesions caused by unresectable malignant tumors that metastasized from various primary organs.

## Materials and Methods

### Patients

Tumors exposed on the body surface, such as unresectable skin cancer and skin metastases from other organ cancers, were targeted. This retrospective study included nine patients who were treated for skin-infiltrating lesions caused by unresectable malignant tumors at the department of dermatology at our university hospital from April 2008 to December 2019. The study consisted of six patients with breast cancer, one with thyroid cancer, one with bladder cancer, and one with basal cell carcinoma of the nose. There were two males and seven females with a mean age of 66.5 years (range of 31–102 years). Patients with insufficient medical records were excluded. This study protocol was approved by the institutional ethical review board for epidemiological and clinical studies at our university.

The need for informed consent by the patients was waived by the ethics committee owing to the retrospective nature of the study.

### Modified Mohs' technique

The original Mohs' paste consists of stibnite (antimony ore ground), *Sanguinaria canadensis* (powdered root of the bloodroot plant), and saturated zinc chloride solution. Although stibnite and *S. canadensis* are used as vehicles due to their suitable viscosity, these products are not available in Japan. The paste was adjusted and modified using zinc oxide powder and glycerin instead.^3–6^ The term “modified Mohs' technique” was used to represent the use of Mohs' paste as palliative treatment that was intended to improve the patient's QOL. First, distilled water and zinc chloride were taken in a beaker and stirred until homogenized (50 mL saturated zinc chloride aqueous solution). To avoid lumps, zinc oxide starch powder (30 g) was dissolved little by little in the solution. Glycerin (15 mL) was added to adjust the viscosity of the paste. The hardness of the paste was adjusted by the amount of zinc oxide starch powder and glycerin (Mohs' made-up paste). White petroleum jelly (Vaseline) was applied to the normal skin around the tumor. Mohs' paste was applied to the tumor site to a thickness of 1 mm and covered with gauze. After 24 hours, the Mohs' paste was removed, and the fixed tissue was removed. The mentioned procedure was performed once or twice per week. Until 2009, the application time for the Mohs' paste was 24 hours. However, from 2010 to 2019, the application time was changed to less than one hour. The paste was thoroughly washed off to reduce the patient's pain. Mohs' treatment was performed once every one to two weeks at a medical facility. Modified Mohs' technique is accompanied by a risk of bleeding due to arterial collapse. Arterial embolization was performed to reduce potential hemorrhaging.

### Topical application of zinc oxide powder

**FIG. 1.**
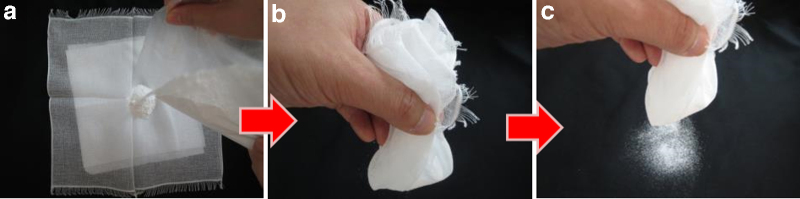
Topical application of zinc oxide powder. **(a)** Place zinc oxide powder on top of one piece of gauze, **(b)** wrap the gauze around the zinc oxide powder, and **(c)** shake the powder on the entire affected area until completely white, apply gauze, and secure with tape.

**FIG. 2.**
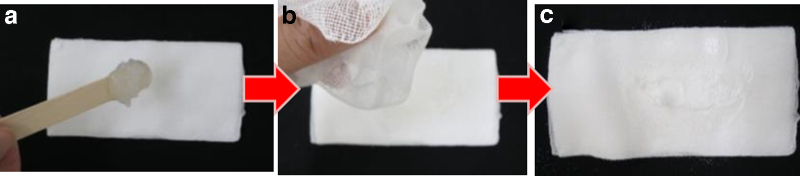
Topical application of zinc oxide powder for difficult lesion locations. **(a)** Apply Vaseline to a piece of gauze, **(b)** add zinc oxide powder on the Vaseline-coated area, and **(c)** apply this gauze to the affected area and secure with tape.

The zinc oxide powder was a 1:1 mixture of zinc oxide and potato starch. Figure 1 shows the general topical application procedure for the zinc oxide powder. Figure 2 shows topical application of zinc oxide powder for lesions that are difficult to treat, such as those close to the nasal cavity or the oral cavity.

## Results

Table 1 gives the detailed characteristics of the nine patients. Arterial bleeding was observed in two cases, oozing in six cases, exudates in nine cases, and malodor in eight cases. There were eight patients with raised skin lesions and one patient with ulcerated lesions. From the medical record description, the doctor and the patient compared the relief of symptoms such as bleeding, exudate, and malodor, that is, the improvement of patients' QOL before and after. Until 2011, only the modified Mohs' technique (cases 2 and 3) was performed. The tumor size, bleeding, exudates, and malodor were controlled by the Mohs' paste. Starting 2012, zinc oxide powder (cases 4, 5, 6, 7, and 8) was applied topically. There was no pain, the malodor and exudate were alleviated, and no ulcers formed even if the powder adhered to normal skin. The burden of treating skin lesions was significantly reduced for the patients and their families because they were able to perform self-treatment in a short time (within a few minutes) at home. For arterial hemorrhage (cases 1 and 9), arterial embolization was performed in the department of radiology before the modified Mohs' technique. In case 1, a feeding blood vessel of the tumor was observed from the thyroid artery, which is a branch of the external carotid artery. Arterial embolization was performed on the feeding blood vessel of the tumor.

**Table 1.** Patient Characteristics

Case	Age	Gender	Underlying disease	Site of skin metastases	Size (cm)	Pretreatment	Symptoms	Treatment	Side effect	Effects
1	78	M	Thyroid cancer	Anterior neck	13 erio × 7	B	Arterial Bleeding Malodor Exudate	E: four times in total/5M, MM: 12 times in total/1–2W (24 hours)	Increased blood loss due to Mohs' paste, surrounding skin ulcers	Embolization reduced bleeding, Mohs' paste reduced tumor size, zinc oxide starch powder reduced malodor and exudate
2	51	F	Left breast cancer	Left front chest to back	18 t fr × 2	S	Bleeding Malodor Exudate	MM: seven times in total/2W (45 minutes–1 hour)	Pain	Bleeding:↓ Exudate:↓ Malodor:↓
3	39	F	Right breast cancer	Sternum	5 × 5 Ulcer	S	Exudate	MM: two times in total/2W (45 minutes–1 hour)	None	Bleeding:↓ Exudate:↓ Malodor:↓
4	70	F	Right breast cancer	Whole precordial area	17 le p × 1	S	Bleeding Malodor Exudate	Z: 3W every day	None	Bleeding:↘ Exudate:↓ Malodor:↓
5	80	F	Left breast cancer	Left front chest to back	8 × 7 × 1	S	Bleeding Malodor Exudate	Z: 6M every day	None	Bleeding:↘ Exudate:↓ Malodor:↓
6	77	F	Left breast cancer	Left precordial area	5 × 6 × 3	S	Bleeding Malodor Exudate	Z: 7M every day	None	Bleeding:↘ Exudate:↓ Malodor:↓
7	31	F	Right breast cancer	Right chest	13 ht c × 3	S	Bleeding Malodor Exudate	Z: 3M every day	None	Bleeding:↘, Exudate:↓ Malodor:↓
8	102	F	Basal cell carcinoma	Nose	4 × 4	S	Exudate Malodor	Z: 4M every day	None	Exudate:↓ Malodor:↓
9	71	M	Bladder cancer	Rear of left thigh	12 r of × 2	B	Arterial Bleeding Malodor Exudate	E: two times in total, MM: five times in total/1–2W, Z: 3M every day	None	E: bleeding: ⇊ MM: tumorsize:↓ Z: malodor:↓ Exudate:↓

B, bacitracin fradiomycin sulfate; E, artery embolization; F, female; M, male; MM, modified Mohs technique; S, sulfadiazine; Z, topical application of zinc oxide starch powder; ↓, alleviation; ↘, slightly reduced; ⇊, considerably reduced.

In case 9, four arteries feeding the skin metastatic lesion on the outside of the left thigh were embolized. After the modified Mohs' technique was performed in these latter cases, the exudate and malodor were controlled; however, bleeding reoccurred. Therefore, arterial embolization was performed a total of four times in case 1 and two times in case 9, after which the tumor size, bleeding, exudate, and malodor were controlled.

In case 1, the paste adhered to the normal skin surrounding the lesion and skin ulcers formed due to continuous fixation for 24 hours. After the application time was changed to less than one hour, no skin ulcerations were observed. Patients with raised masses rarely complained of pain with the modified Mohs' paste; only one patient with ulcerative lesions complained of considerable pain. In case 8, no fistula was formed in the floor of the mouth, and the powder did not flow into the trachea when Vaseline was used for topical application of the zinc oxide.

Table 2 gives a comparison between the modified Mohs' technique and topical application of zinc oxide powder. Our cases indicated that topical application of zinc oxide powder had less hemostatic and tumor-reducing effects than the modified Mohs' technique. Both the modified Mohs' technique and zinc oxide powder application were effective in reducing malodor and exudate. In addition, topical application of zinc oxide powder was pain free and did not cause ulcers on normal skin. Unfortunately, all cases died of the primary disease.

**Table 2.** Comparison of the Modified Mohs' Technique and Topical Application of Zinc Oxide Powder

	MM	Z
Enforcement	Available only at medical institutions	Possible at home
Occurrence	Every —one to two weeks	Every day
Treatment time	Within one hour	Within minutes
Clinical requirements	Need to prepare Mohs' paste	None (commercial item)
Pain	Severe	None
Effects on normal skin	Ulceration (+)	Ulceration (−)
Hemorrhage	Alleviation	Slightly reduced
Malodor	Alleviation	Alleviation
Exudate	Alleviation	Alleviation
Tumor shrinking effect	Reduction	Slightly reduced
Contraindications	Arterial bleeding, lesions that may flow into the oral cavity, nasal cavity, and respiratory tract	None

MM, modified Mohs technique; Z, topical application of zinc oxide starch powder.

## Discussion

In this study, we reviewed the modified Mohs' technique and topical application of zinc oxide powder as part of palliative treatment for skin lesions caused by unresectable malignant tumors. This is the first study to compare a 1-hour application of Mohs' paste with the topical application of zinc oxide powder versus the 24-hour application of Mohs' paste. The modified Mohs' technique appeared to be superior in cases wherein the tumor was proliferating. However, the topical application of zinc oxide powder alone was as effective as the modified Mohs' technique in reducing malodor and exudate from the skin lesions. Therefore, the modified Mohs' technique should be used for tumor reduction and bleeding control, whereas topical application of zinc oxide powder should be used for reducing malodors and exudates. In case 9, the modified Mohs' technique and topical application of zinc oxide powder were used in combination. Furthermore, a combination of both methods may substantially improve the patient's QOL.

Although the original method of Mohs' chemosurgery was developed to remove skin tumors, in this study, Mohs' paste was used as a palliative treatment to improve the patient's QOL. Modified Mohs' technique has been reported as an effective treatment to decrease bleeding, decrease frequency of wound dressing changes, decrease odor and degree of pain experienced, and increase the patient's QOL.^3–5^ Similarly, its efficacy has been demonstrated through significant decreases in tumor size and the number of dressing changes required.^6^ No guiding philosophy exists regarding the use interval and contact time with Mohs' paste; the progress and depth of consolidation depend on contact time. Fukaya et al. reported that 10 mm depth of the tumor surface was fixed in 24 hours when using 1-hour treatment with the modified Mohs' paste.^6^ However, problems, such as changes in hardness and viscoelasticity with time and liquefaction by exudate, occur. Tsuruta et al. reported a mixture of Mohs' paste and zinc oxide 10% single ointment, which showed that the viscosity of modified Mohs' paste could be adjusted with glycerin.^7^ The effect of prescription improvement of the Mohs' paste on the pharmacological effect was examined with reference to water absorbing property, and its tumor tissue invasion fixation depth as an indicator.^8^ In the modified Mohs' technique, invasion fixation depth increased depending on application time.^7^ The intended purpose of Mohs' paste application is to determine the appropriate use interval and contact time. The original Mohs' chemosurgery used a contact time of 24 hours for the purpose of removing the tumor. However, it has been reported that a contact time of 5–30 minutes is sufficient to prevent oozing from the tumor surface.^3–5^ Patients often experienced severe pain and inability to tolerate the 24-hour application of the Mohs' paste; thus, by reducing the application time of modified Mohs' technique to one hour, the patients' pain was relieved. Pain control in the Mohs' paste was achieved by the administration of analgesics, the topical use of petroleum jelly on the surrounding healthy skin to protect it from the paste, and a reduction in the contact time of the Mohs' paste to the skin.^3,6^ Several techniques have been used for containing the oil, such as thick application of petroleum jelly or affixing dressing agents.^3–5^ When using Mohs' paste, it is important to protect the normal healthy skin. In another trial, simple zinc oxide ointments were mixed with Mohs' paste in a 1:1 ratio to improve the viscosity for avoiding the infection in surrounding organs and pain.^9^ Fukaya et al. reported that the treatment period and repetition of treatment depended on factors, such as the cooperation of the patients, and tumor characteristics, such as the size and frequency and duration of bleeding. Although the frequency of the modified Mohs' technique was different for each case (430 days, with a median of 7 days), application once a week appears to be effective.^6^ One hour is considered an optimum duration for the modified Mohs' technique.^6^ The Mohs' method was time consuming, and the procedure required a medical facility. The Mohs' paste also cannot be stored for any length of time due to a rapid loss of its adhesive properties. These factors limit the application of this method as compared with the application of topical zinc oxide. Patients were able to perform self-treatment with zinc oxide powder, which considerably reduced the clinical and personal treatment burden. Heat dissolution will be generated with 50 mL of saturated solution of zinc chloride. Therefore, we focused on zinc oxide powder, which is one of the ingredients of Mohs' paste.

Zinc oxide is a very old drug. There are few detailed research reports on the mechanism of action of the drug itself. Zinc oxide binds to or adsorbs on the skin to form insoluble precipitates and films, which have astringent, anti-inflammatory, protective, and mild antiseptic effects. It also reduces the permeability of capillaries and suppresses plasma leaking and leukocyte emigration, which has the effect of suppressing inflammation and drying the surface of the wound or ulcer.^10,11^

Breast cancer is the malignancy that is most likely to develop malignant wounds.^3^ Bleeding from breast cancer can occur in palliative care patients with terminal stage disease and during neoaduvant chemotherapy or radiation therapy for advanced cancer with skin invasion. Suturing or electric coagulation has poor rates of successful hemostasis and can even cause further bleeding because the malignant wounds of breast cancer tend to be friable and contain many abnormal small vessels.^3^ In this study, it was not just breast cancer patients but also the patients with thyroid cancer and bladder cancer who showed the same tendency. In patients with abundant tumor vascularization, it is contraindicated to perform the modified Mohs' technique before embolization because of the increased risk of massive bleeding. The risk of hemorrhage due to the rupture of the external carotid artery while using Mohs' chemosurgery for a parotid tumor has been reported, and caution is required depending on the site of use of the Mohs' paste.^12^ Multidisciplinary treatments, including embolization, for bleeding are required. The modified Mohs' technique is also contraindicated for lesions that may enter the oral cavity, nasal cavity, and respiratory tract. Like some other methods of hemostasis, alginate dressings, local tranexamic acid, or adrenaline-soaked gauze are validated topical treatments.^13^ Oral antifibrinolytics can be considered to prevent further bleeding.^13^ Embolization or radiotherapy may be considered according to overall patient attitude.^13^ Later application of metronidazole powder is helpful for a more complete deodorization.^3^

One of the major limitations of this study is the number of patients in each group and the fact that patient 9 received both the Mohs' and the zinc oxide treatments. Case 9 cannot be used in a comparison if both treatments were performed. The doctor and the patient subjectively evaluated the effects of bleeding volume, degree of pain, reduced exudates and malodors, and tumor shrinkage with similar results. However, it lacks objective measurements of pain and the patient's QOL, as well as measurements of exudate and malodor. There are no statistical analyses for any of the results. This needs to be confirmed by larger clinical studies or large multicenter retrospective studies to increase number of patients and enable statistical evaluation of the data. Small observational studies can provide important data that can lead to larger confirmatory studies.

## Conclusion

Skin-infiltrating lesions caused by unresectable malignant tumors eventually self-destruct and present as large ulcers. Symptoms, such as bleeding, exudate, pain, and malodor, significantly worsen the patient's QOL. In such cases, the modified Mohs' technique and topical application of zinc oxide powder were useful as palliative treatments and improved the patient's QOL significantly. After arterial embolization if the tumor is highly vascular, the modified Mohs' technique and topical application of zinc oxide powder can decease exudate, malodor, and bleeding;, it has a tumor shrinkage effect in some patients with unresectable dermal metastases and seems to work better on raised rather than ulcerated lesions. The effective management of skin ulcers caused by unresectable malignant tumors can be achieved by properly using the modified Mohs' technique and by topical application of zinc oxide powder. The role of the dermatologist is important to ensure proper usage of both treatments.

## Abbreviation Used

QOL: quality of life
